# Lung Metastasis after an Eighteen-Years-Long Disease-Free Period since Uterine Leiomyosarcoma Diagnosis

**DOI:** 10.1155/2014/961675

**Published:** 2014-03-12

**Authors:** M. Guazzaroni, D. Tosti, M. Rascioni, M. Mataloni, D. Citraro, G. Simonetti

**Affiliations:** Department of Diagnostic Imaging, Molecular Imaging, Interventional Radiology and Radiation Therapy, University Hospital Tor Vergata, Viale Oxford, 81-00133 Rome, Italy

## Abstract

Uterine leiomyosarcoma (ULMS) is an uncommon malignancy that accounts for one-third of uterine sarcomas and represents 1% of all uterine malignancies, with an incidence averaging 0.5–1/100,000/year. The prognosis is poor due to its intrinsic aggressiveness and its characteristic high metastatic potential with reported distant metastatic spread in lung, abdomen, soft tissue, and brain. We present the case of a 67-year-old woman with lung metastasis after eighteen years since uterine leiomyosarcoma diagnosis and its following surgical resection. The diagnosis of pulmonary metastases was obtained by reviewing the histology of the previous uterine tumor: the tumor cells were immunoreactive for CD10, PR, and smooth muscle actin (SMA), but negative for desmin, S100, CD34, CD 117, cytokeratins AE1AE3, CD68R, and ER. To our knowledge, this disease-free interval is the longest among previous reports of pulmonary metastasis of uterine leiomyosarcoma.

## 1. Introduction

Uterine leiomyosarcoma (ULMS) is an uncommon aggressive uterine cancer characterized by a poor prognosis. Although these tumors are usually confined to the uterus at the time of diagnosis, there is a high incidence of recurrence. Often, patients show recurrency within 2 years from the initial diagnosis, and almost the total of these ones, in a percentage quantifiable in about 90%, develops distant metastases both alone or in association with pelvic recurrence [1]. ULMS usually metastasizes to the lungs, peritoneal cavity, and vagina, followed by the retroperitoneum, liver, and bone.

The initial treatment of choice of uterine leiomyosarcoma is the surgical resection and, in addition to this, adjuvant therapy (including chemotherapy and radiotherapy) can be used to reduce the risk of recurrence, despite the fact that the clinical efficacy is still uncertain [2].

## 2. Case Presentation

In January 2013, a 67-year-old woman, with history of uterine leiomyosarcoma, was admitted to our department for chest discomfort. Her clinical data reported a total abdominal hysterectomy and right salpingo-oophorectomy due to the uterine tumor measuring 80 mm, both performed in 1995. The resected tumor was histologically identified as low-grade leiomyosarcoma. In fact, microscopically, it presented spindle cells with marked cytological atypia, necrosis, and a high mitotic index. The tumor cells were immunoreactive for CD10+, PR, and smooth muscle actin (SMA), but negative for desmin, S100, CD34, CD117, cytokeratins AE1AE3, CD68R, and ER. Staging imaging, performed through total body contrast enhanced CT (CECT) scan, was negative for metastatic localizations. After surgical intervention, an adjuvant chemotherapy with doxorubicin was administered to the patient. Clinical and radiological followup showed no evidence of metastatic disease prior to the current presentation.

**Figure 1:**
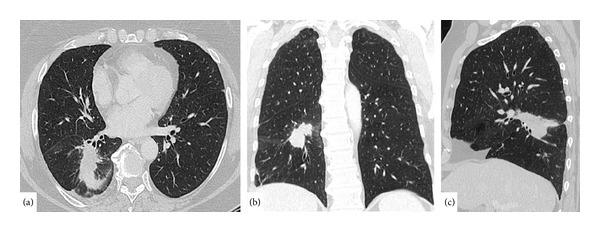
Axial, coronal, and sagittal CT images showing mass lesion in the inferior lobe of right lung.

In January 2013, the patient came to our institute because of chest discomfort. A chest radiograph was performed and a right lung lesion was detected. Therefore a CECT confirmed a tumor mass of 35 mm in size in her inferior lobe of right lung (Figure 1). No other distant metastases or local recurrence was found.

**Figure 2:**
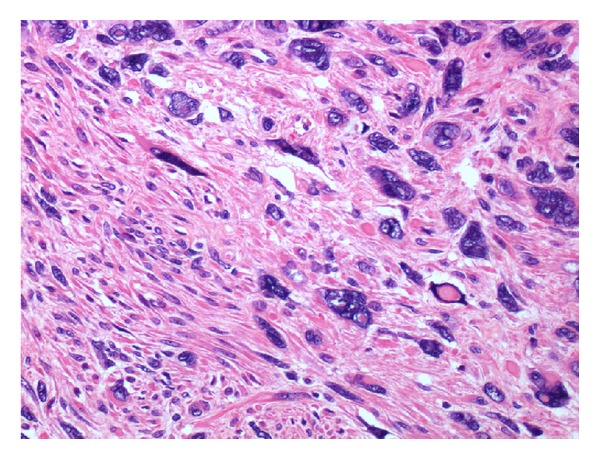
H&E section shows cellular smooth muscle tumor with nuclear pleomorphism.

**Figure 3:**
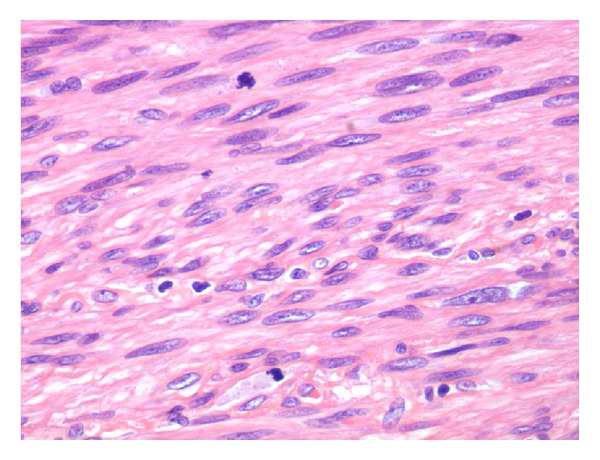
H&E section. Atypical mitoses.

**Figure 4:**
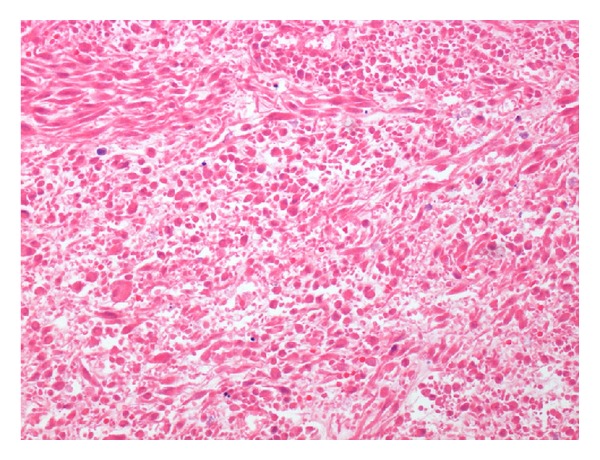
H&E section. Necrosis.

In February 2013, a right lower lobectomy was realized through anterolateral thoracotomy. The lesion was sent to our Department of Anatomic Pathology for histopathological examination: macroscopically, the nodule showed infiltration of the right bronchial tubes until 0.5 cm from bronchial resection margin. Microscopically, there were numerous atypical mitoses and extensive areas of necrosis (Figures 2, 3, and 4).

Immunohistochemical studies showed that the neoplastic cells were SMA+, CD10+, and PR+, while they were negative for desmin and keratin. These results were compared with the histological data of the previous tumor, and a final diagnosis of pulmonary metastasis was obtained: the immune histochemistry supported the diagnosis of metastases of leiomyosarcoma.

Restaging PET-CT brain, thorax, abdomen, and pelvis were negative for local recurrence or distant metastases.

## 3. Discussion 

Uterine leiomyosarcoma (ULMS) accounts for 1/3 of uterine sarcomas and represents 1% of all uterine malignancies, with an incidence of 0.5–1/100,000/year. Although 60% of patients present with disease confined to the uterus, cure rates range from 20 to 60%. For those patients with locally confined disease, the long-term disease-free survival is close to, or below, 50% but is definitely lower than 10% for those with advanced stage disease [3]. ULMS have high metastatic potential: patients with metastatic disease exhibit a dismal prognosis and, except for a subset of patients with limited and completely resectable disease, the median survival is less than 1 year. More frequent documented sites of metastatic localizations are lungs, abdomen, soft tissues, and brain, while less commonly metastases can involve the breast and bone [4, 5].

This case confirms that the leiomyosarcoma is a high-grade malignant tumor with recurrency that can arise even after long periods of dormancy. In fact we observed the appearance of lung metastasis after 18 years from the diagnosis of uterine leiomyosarcoma. To our knowledge, this disease-free interval is the longest among previous reports of pulmonary metastasis of uterine leiomyosarcoma. Kato and Kanauchi [6] described a case of lung metastasis of uterine leiomyosarcoma after fifteen years, reported as the longest disease-free interval. Previously, there have been reports of lung metastases after 7 years by Funada et al. and Laffargue et al. [7, 8].

The initial treatment of choice of uterine leiomyosarcoma is surgery. The use of adjuvant chemotherapy has limited impact on clinical outcome. The overall recurrence rate and disease-free survival were similar in patients who receive adjuvant treatment and in those who do not. Doxorubicin, gemcitabine, and gemcitabine plus docetaxel are treatment options in women with inoperable, locally advanced, recurrent, or metastatic uterine LMS as first- or second-line therapy. Radiation therapy appears to improve local control without any significant impact on survival because most recurrences involve distant sites [9].

Careful followup after treatment of the uterine leiomyosarcoma is recommended because metastatic leiomyosarcoma can possibly appear even after a long interval, and its progress can be so intensive that adjuvant chemotherapy is necessary if it cannot be treated surgically alone.
